# Characteristics, Treatment Patterns and Survival of International Federation of Gynecology and Obstetrics Stage IV Epithelial Ovarian Cancer—A Population-Based Study

**DOI:** 10.3390/cancers15235676

**Published:** 2023-11-30

**Authors:** Dorothee Jakob, Claudia Schmoor, Raphael Reuten, Marie Louise Frevert, Dominik Dannehl, Lina Jansen, Silke Hermann, Peter Jungmann, Andreas Daniel Hartkopf, Ingolf Juhasz-Böss, Florin Andrei Taran

**Affiliations:** 1 Department of Obstetrics and Gynecology, University Medical Center Freiburg, 79106 Freiburg, Germany; dorothee.jakob@uniklinik-freiburg.de (D.J.); raphael.reuten@pharmakol.uni-freiburg.de (R.R.);; 2 Clinical Trials Unit, University Medical Center Freiburg, Faculty of Medicine, University of Freiburg, 79110 Freiburg, Germany; 3 Institute of Experimental and Clinical Pharmacology and Toxicology, Medical Faculty, University of Freiburg, 79110 Freiburg, Germany; 4 Department of Women’s Health, Tuebingen University Hospital, 72076 Tuebingen, Germanyandreas.hartkopf@med.uni-tuebingen.de (A.D.H.); 5 Epidemiological Cancer Registry of Baden-Württemberg, German Cancer Research Center (DKFZ), 69120 Heidelberg, Germany

**Keywords:** ovarian cancer, survival, surgery, cancer-directed therapy

## Abstract

**Simple Summary:**

Ovarian cancer (OC) is the most lethal gynecologic malignancy, with a relative 5-year survival rate of between 40% and 50%. We used the Cancer Registry of Baden-Württemberg to identify characteristics, treatment patterns and survival of OC patients with International Federation of Gynecology and Obstetrics (FIGO) stage IV who were registered over a period of 8 years (2012–2019). The aim of the present analysis was to describe an unselected patient population with primary diagnoses of FIGO stage IV OC with respect to baseline patient and tumor characteristics, treatment strategies and prognosis in terms of overall survival. In this cohort of patients with FIGO stage IV OC, more than 80% of the patients received cancer-directed treatment. Age and high-grade serous histology were determinants for survival. The highest overall survival rate was observed in patients who underwent surgery followed by systemic treatment.

**Abstract:**

Background: The aim of the present study was to describe an unselected population of patients with diagnosis of FIGO stage IV OC. Methods: Data from 1183 patients were available for analysis. Results: The majority of patients (962/1183, 81.3%) received cancer-directed treatment. The median follow-up time was 3.8 years, and the median overall survival duration was 1.9 years. Notably, patients >80 years had a low overall survival rate (HR of age >80 years vs. ≤50 years was 3.81, 95%-CI [2.76, 5.27], *p* < 0.0001). The survival rate was best in patients with HGSOC (*p* < 0.0001). The highest overall survival rate was observed in patients in the group with surgical intervention followed by systemic treatment, with an unadjusted HR of 0.72, 95%-CI [0.59, 0.86], *p* = 0.007 vs. systemic treatment only. After adjustment for age and histology, survival differences between treatment schemes were smaller (HR 0.81, 95%-CI [0.66, 1.00], *p* = 0.12). Conclusions: In this cohort of patients with FIGO stage IV OC, more than 80% of the patients received cancer-directed treatment. Age and high-grade serous histology were determinants for survival. The highest overall survival rate was observed in patients who underwent surgery followed by systemic treatment.

## 1. Introduction

In Germany, more than 7000 women are diagnosed with ovarian cancer (OC) every year, and 5400 women die due to OC. OC is the most lethal gynecologic malignancy, with a relative 5-year survival rate of between 40% and 50% [1]. Median survival duration for patients with International Federation of Gynecology and Obstetrics (FIGO) stage IV OC ranges from 15 to 29 months [2]. One of the main prognostic factors in advanced OC is the presence of macroscopic residual disease after cytoreductive surgery [3,4,5].

Patients with FIGO stage IV OC, by definition, have the most extensive tumor burden and therefore often need multivisceral resections, which are associated with high perioperative and postoperative morbidity and mortality [6,7]. Serious complications arise in 11–22% of patients undergoing radical cytoreductive surgery [8,9,10]. If patients are not eligible for primary surgery due to inoperable disease extent or reduced performance status, neoadjuvant chemotherapy followed by interval debulking surgery is recommended [11,12,13]. Although neoadjuvant chemotherapy increases the number of patients with postoperative R0 status, it remains unclear whether this treatment option is equivalent to primary surgical treatment with respect to oncologic outcomes [14,15,16,17].

Treatment recommendations for patients with FIGO stage IV OC are based solely on studies that have mostly included FIGO stage III OC patients [18,19,20]. Only a small number of retrospective studies directly investigate the benefit of radical surgery to achieve R0 resection in FIGO IV OC [21]. Furthermore, it is unclear how guideline recommendations are implemented into the daily routine for patients with FIGO stage IV OC [22]. Similarly, only a few studies have focused on characterizing the patient population with FIGO stage IV OC. Thus, the integration of cancer registry data into clinical cancer research is of increasing interest.

Baden-Württemberg is the third largest federal state in Germany, with a population of 11 million inhabitants, representing approximately 13% of the German population [23]. Beginning in 2009, a legal obligation to register all pre-malignant and malignant diagnoses within the Baden-Württemberg Cancer Registry was introduced [24]. We used the Cancer Registry of Baden-Württemberg to identify characteristics, treatment patterns and survival of OC patients with FIGO stage IV who were registered over a period of 8 years (2012–2019). The aim of the present analysis was to describe an unselected patient population with primary diagnoses of FIGO stage IV OC with respect to baseline patient and tumor characteristics, treatment strategies and prognosis in terms of overall survival.

## 2. Methods

We conducted a data inquiry and analysis in collaboration with the Cancer Registry of Baden-Württemberg (CRBW). The CRBW provided to the investigators anonymized data on individual patients who met the inclusion criteria, for further analysis. Therefore, in accordance with German law, approval by an ethics committee and informed consent requirements were not applicable. All patient data were anonymized by the CRBW. In practice, this meant that all information which might allow conclusions to be drawn about an individual patient or a specific hospital was eliminated by the CRBW for data protection reasons. Consequently, age was only available in 5-year categories; for dates of diagnosis and treatments, only the month and the year were given; and follow-up and survival times were given in days, without the exact dates of last follow-up or death being provided.

Patients diagnosed with primary OC and FIGO stage IV at the time of diagnosis (International Statistical Classification of Diseases and Related Health Problems (ICD)-10: C56) between 1 January 2012 and 31 December 2019 were included in the study. Morphological diagnosis was documented within the CRBW by ICD-O-3 [25]. If no FIGO classification was documented, the patient was categorized as FIGO stage IV OC based on the presence of a tumor that had spread outside the abdominal cavity (including malignant pleural effusion) and/or visceral metastases and any distant metastasis (including lymph nodes). We only considered a patient’s first OC diagnosis and excluded patients with missing tumor stage information and patients for whom only a death certificate was issued (DCO). The following variables were extracted from the CRBW: age, Eastern Cooperative Oncology Group (ECOG) performance status, histology, metastatic sites, treatment data (surgical therapy (including intention of the surgical procedure), systemic therapy and radiation therapy), residual disease after surgery, and follow-up with respect to overall survival.

For comparison of overall survival rates between initial treatment strategies, treatment groups were defined by the initial treatment given, irrespective of subsequent treatments. Patients were allocated to the “surgery group” if surgery was the primary treatment and was performed within 2 months after diagnosis of OC. Patients were allocated to the systemic group if systemic therapy was the primary treatment and started within 2 months after OC diagnosis. A further comparison was performed for different treatment schemes. Patients were allocated to the “surgery-only group”, if surgery was performed within 2 months post-diagnosis and no further systemic therapy was administered for at least 3 months thereafter. Patients were assigned to the “surgery followed by systemic therapy group” if surgery was performed within 2 months post-diagnosis and systemic therapy was started within 3 months thereafter. Patients were classified as belonging to the “systemic group” if systemic therapy was started within 2 months post-diagnosis and follow-up was available for at least 3 months. The restrictions regarding the minimum follow-up time after primary treatment were necessary to avoid any time-dependent bias.

Descriptive analyses were performed by calculating absolute and relative frequencies for categorical data and by calculating means and ranges for continuous data. The overall survival time was calculated from the point of primary diagnosis to death. The median follow-up time was calculated by the reverse Kaplan–Meier estimator.

Survival probabilities for the patient cohort and of subgroups of patients defined by patient and tumor characteristics at baseline and by treatment group were estimated by the Kaplan–Meier method. Additionally, survival rates by treatment group, adjusted for age and histology, were calculated using multivariate Cox regression models. Differences or similarities between treatment groups were interpreted as descriptive rather than causal because of possible confounding resulting from the retrospective nature of the study. Univariate and multivariate comparisons between patient groups were performed using Cox regression models, in which hazard ratios were calculated with 95% confidence intervals (CI), and *p*-values, which were interpreted in a descriptive instead of a confirmatory sense.

## 3. Results

**Figure 1:**
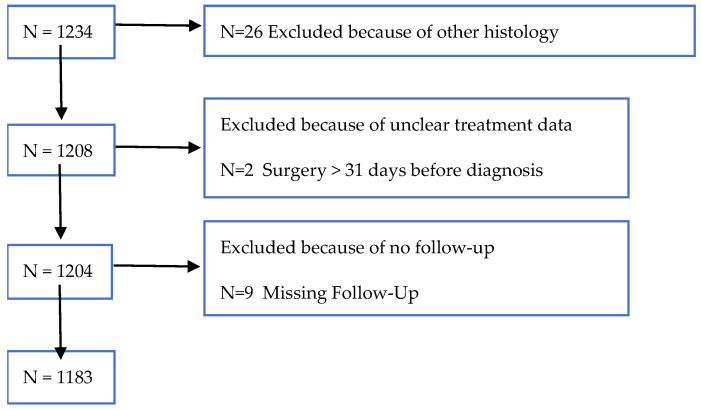
Flow chart of patient inclusion and exclusion.

Between 1 January 2012 and 31 December 2019, 7306 patients with OC were registered in the CRBW, of which 1234 patients met our inclusion criteria for FIGO stage IV OC. Of these, fifty-one patients were excluded due to histologies other than OC, unclear treatment data, or missing follow-up. Subsequently, data of 1183 patients with FIGO stage IV OC were available for analysis (Figure 1).

The main characteristics of the patient cohort are displayed in Table 1. The majority of the patients were between 66 and 80 years old; however, there were significant proportions of patients younger than 50 years (8.8%) and older than 80 years (15.2%) (Table 1). The most frequent metastatic sites were pleura (including pleural effusion) (309 patients, 32.4%), liver (278 patients, 29.1%) and lymph nodes outside the abdominal cavity (225 patients, 26.7%) (Table 1).

Table 2 shows the documented data on patients’ basic treatment: for 221 patients (18.7%), no report of treatment for FIGO stage IV OC (Table 2) was submitted to the CRBW. The summary of treatment strategies shows that for 669 patients (56.6%), treatment included any kind of surgery; for 881 patients (68.6%), a treatment with systemic therapy was reported, and for 523 patients (44.2%), a treatment with both surgery and systemic treatment (Table 2) was reported.

Of the 669 patients who received surgery, 511 patients (76.4%) underwent surgery once, 125 patients (18.7%) received surgery twice, and three or more surgical procedures were reported in 33 cases (4.9%). Among the 881 patients who received systemic therapy, 403 patients (49.7%) received one line, 316 patients (39.0%) received two lines of systemic therapy, and three or more systemic therapy lines were reported in 92 cases (11.3%) (Table 2).

Documentation on residual status after first surgery was available for 423 patients (63.2%) and RX/unknown was associated with 246 patients (36.8%). Macroscopic residual status comprised macroscopic complete resection (R0) for 157 patients (37.1%), incomplete resection (R1, small residuals 1–10 mm) for 118 patients (27.9%), and incomplete resection (R2, residual disease >1 cm) for 148 patients (35.0%) (Table 2).

Table 3 presents a stratification of patient characteristics based on the received sequence of treatments. Patients older than 80 years and patients with a poor performance status were more likely to receive neither surgery nor systemic treatment, or to receive only one of the two treatments—either surgery only or systemic treatment only. Patients with HGSOC were mostly treated by surgery followed by systemic therapy (42.5%), whereas patients with unspecified histology received no treatment (39.3%) or solely systemic therapy (37.8%) (Table 3).

The initial treatment strategy within 2 months after diagnosis of OC was surgery for 577 patients, and systemic for 305 patients. Treatment groups defined by the treatment scheme started within 2 months after diagnosis of OC were surgery-only for 119 patients, surgery followed by systemic for 376 patients, and systemic for 267 patients (Table 2).

### Survival Analysis

**Figure 2:**
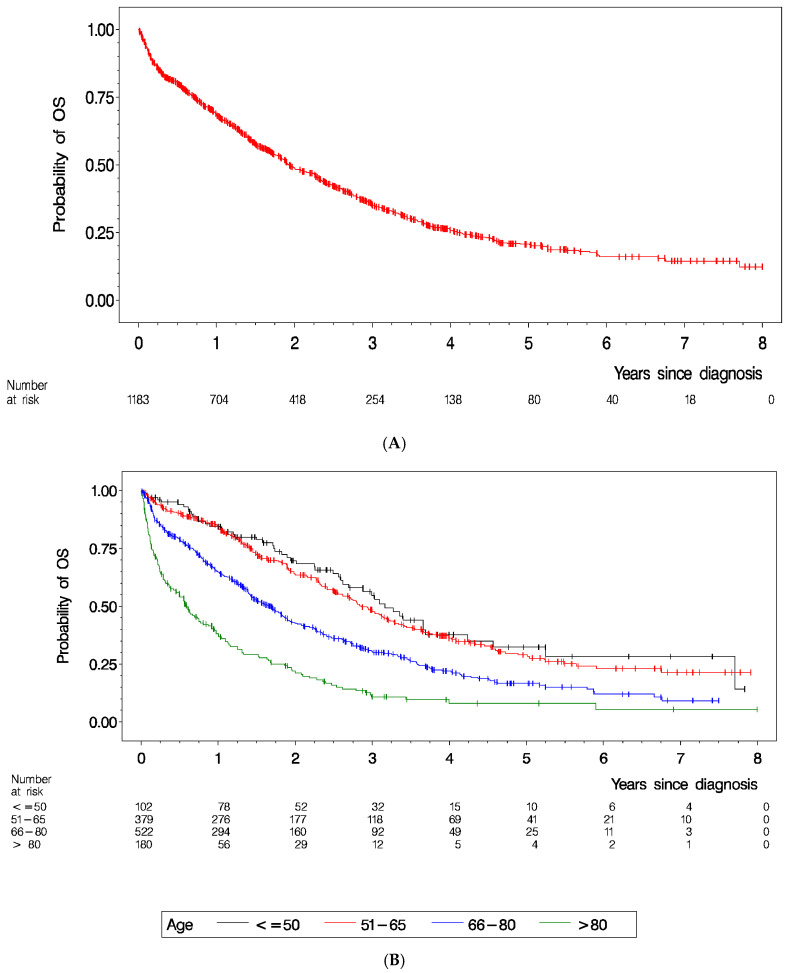

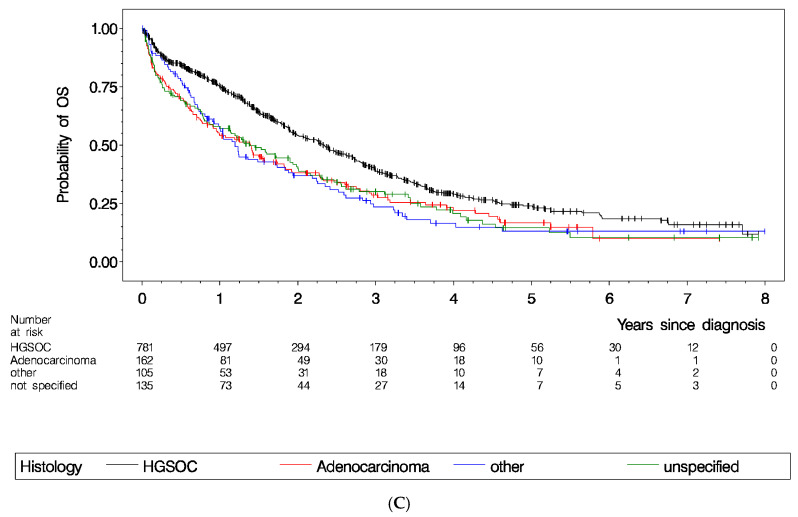
Overall survival probabilities estimated by the Kaplan–Meier method of overall survival: (**A**) entire patient cohort, (**B**) stratified by age, and (**C**) stratified by histology.

**Figure 3:**
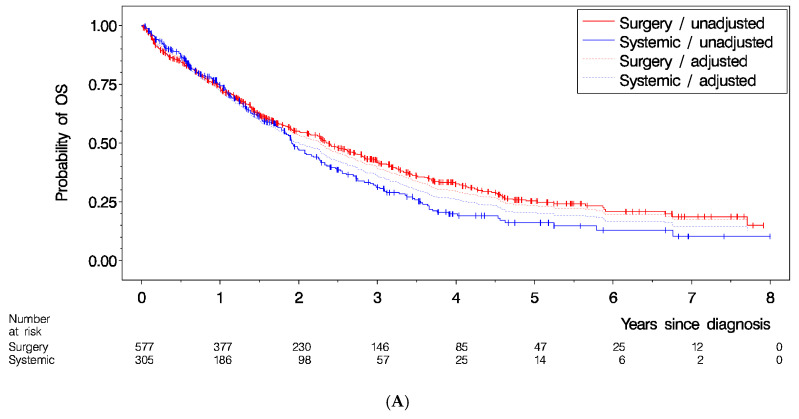

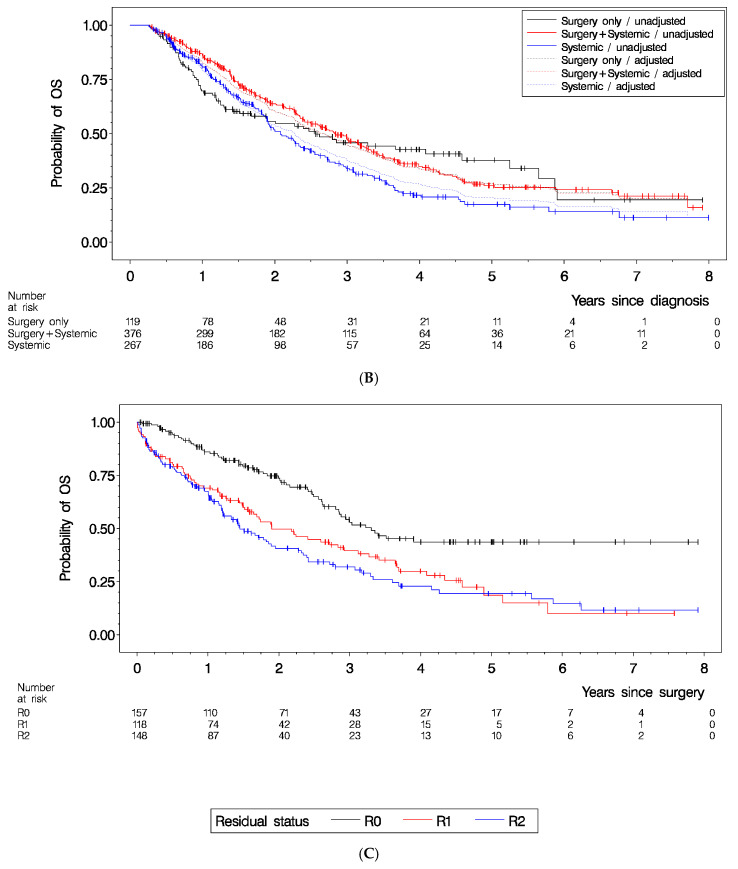
Overall survival by (**A**) primary treatment (unadjusted and adjusted for age and histology), by (**B**) treatment scheme (unadjusted and adjusted for age and histology), and by (**C**) residual status after surgery. (**B**) This analysis only included patients who started treatment within 2 months after initial diagnosis, and for whom the minimum follow-up was at least 3 months after the primary treatment, to avoid any time-dependent bias. (**A**) Primary treatment within 2 months post-diagnosis (N = 882); (**B**) Treatment scheme (N = 762); (**C**) Residual status after first surgery (N = 423) (R0 = macroscopic complete resection, R1 = macroscopic incomplete resection (small residuals 1–10 mm), R2 = macroscopic incomplete resection (residual disease > 1 cm)).

The median follow-up time for the patient population was 3.8 years. At the end of the follow-up period, 434 patients (36.7%) were alive, and 749 patients (63.3%) had died. The overall survival rate of the entire patient cohort is displayed in Figure 2A. Median overall survival time was 1.9 years (interquartile range: 0.7–4.0 years). We analyzed the patient survival as stratified by age, histology and resection status (Figure 2B,C and Figure 3C and Table 4A). Age was a determining factor for survival. In particular, patients >80 years had a low overall survival rate (hazard ratio of age >80 years vs. ≤50 years was 3.81, 95%-CI [2.76, 5.27], *p* < 0.0001) (Figure 2B). Regarding histological subtypes, survival was best for patients with HGSOC (*p* < 0.0001) (Figure 2C).

In an unadjusted analysis, the hazard ratio of primary surgery vs. primary systemic treatment was 0.82, 95%-CI [0.68, 0.97], (*p* = 0.025) (Figure 3A, Table 4B). After adjustment for age and histology, the difference was reduced, with a hazard ratio of primary surgery vs. primary systemic treatment of 0.90, 95%-CI [0.75, 1.08], (*p* = 0.27) (Figure 3A, Table 4B). The highest overall survival rate was observed for patients in the “surgery followed by systemic treatment” group, with an unadjusted hazard ratio of 0.72, 95%-CI [0.59, 0.86], *p* = 0.007 vs. systemic treatment only (Figure 3B, Table 4B). But again, after adjustment for age and histology, the survival differences between treatment schemes were no longer significant (*p* = 0.12) (Figure 3B, Table 4B).

A comparison of the overall survival after first surgery of patients with macroscopic complete resection R0, incomplete resection R1 (small residuals 1–10 mm) and incomplete resection R2 (residual disease > 1 cm) is shown in Figure 3C. The hazard ratio of patients with R1 vs. R0 was estimated as 1.95, 95%-CI [1.38, 2.75], and of patients with R2 vs. R0 as 2.34, 95%-CI [1.69, 3.25], *p* < 0.0001 (Figure 3C).

## 4. Discussion

The present study describes characteristics, treatment patterns and survival of OC patients with FIGO stage IV who were registered at the Cancer Registry of Baden-Württemberg over a period of 8 years. For more than 80% of patients with OC in FIGO stage IV, cancer-directed treatment was documented. Age and high-grade serous histology were determinants for survival. The highest overall survival rate was observed for patients who underwent surgery followed by systemic treatment.

A comparison based on primary treatment within 2 months after diagnosis of FIGO stage IV OC showed a higher survival rate for patients whose primary treatment was surgery than for patients whose primary treatment was a systemic therapy. This is in line with earlier published data [2,19,26,27], and also reflects current treatment recommendations [28]. After adjustments for age and histology, the difference was smaller, but showed the same trend, indicating that primary surgery should be favored. Nevertheless, these results should be interpreted with caution due to selection bias. Younger patients with a good ECOG status and fewer comorbidities are more likely to be selected to undergo extensive surgical procedures for FIGO stage IV OC [2,29]. Data on the ECOG status and on comorbidities were rarely available or not available, respectively, and could therefore not be accounted for in the analysis. Hence, the selection of patients who benefit from debulking surgery is crucial in patients with FIGO stage IV OC [27].

Patients with FIGO stage IV OC represent a vulnerable population with an elevated risk of death. In contrast to the available evidence, we demonstrated that more than 80% of the patients with OC at FIGO stage IV received cancer-directed treatment. Shalowitz et al. described a similar rate of more than 80% of patients with FIGO stage IV OC who received cancer-directed treatment [29]. Zijlstra et al. found a similar rate of 80% of patients with FIGO stage IV OC who received targeted cancer therapy [30]. In both studies, advanced age and FIGO stage IV at initial diagnosis were described as factors associated with not receiving targeted cancer therapy [29,30]. Additionally, in the study by Zijlstra et al., a considerable number of the patients who did not receive cancer-directed treatment were involved in the process leading to this decision; hence, the patient’s choice appears to have been the main reason for the decision to forgo cancer-directed treatment [30]. The second most common reason for not providing cancer-directed treatment was a poor general condition of the patient, which might indicate careful selection of patients for treatment [30].

In the study by Shalowitz et al., 60% of the patients treated by surgery had FIGO stage IV disease [29]. Similarly, our study found that about 50% of patients with FIGO stage IV OC underwent surgery as primary treatment, with the intention of surgery being curative in 55% of the patients with available data on the intention. Furthermore, a macroscopic complete resection was reported in more than one-third of the patients with available data on resection status. The study by van Altena et al. found that treatment strategies in patients with advanced OC have changed over time, with more and more patients receiving neo(adjuvant) chemotherapy and undergoing optimal debulking surgery [31]. In addition, implementation of and adherence to guidelines leads to a change in treatment strategy and an improvement in relative survival in advanced OC [32].

More than 40% of patients with FIGO stage IV OC received both surgery and systemic treatment in accordance with current guideline recommendations [28]. Older age and patient preferences are the most common reasons for omission of guideline-adherent treatment in patients with advanced OC [33]. Importantly, an apparent failure to receive guideline-adherent care does not necessarily indicate an inappropriate treatment plan [32]. For example, some patients with extensive disease or significant comorbidities may survive longer after receiving systemic therapy than after surgical management [29]. Likewise, the decision to pursue surgery is not always clinically beneficial [29].

The strengths of this study include the analysis of a large dataset from the Cancer Registry of Baden-Württemberg which included more than 1100 patients with OC at FIGO stage IV within a time span of 8 years. The dataset is well controlled by independent supervision. Therefore, it is reliable with respect to clinical characteristics, treatment data and outcomes. However, several limitations need to be considered: First, this is a retrospective study, which is an inherent aspect of large-database studies. Second, observational studies are prone to bias from unmeasured confounders which could affect the present results (e.g., genetic status, dose reduction of systemic therapy, and disease progression under treatment). Thus, outcomes deriving from registry settings have limitations and should be interpreted with caution, since treatment strategies for OC and survival of patients with OC are influenced by a multitude of factors. Finally, although our 8-year study period allowed us to examine a large cohort of women, it cannot account for changes in OC management that were implemented during this time span.

## 5. Conclusions

In this large cohort of patients with FIGO stage IV OC, more than 80% of the patients received cancer-directed treatment. Age and high-grade serous histology were determining factors for survival. The highest overall survival rate was observed for patients who underwent surgery followed by systemic treatment.

## Figures and Tables

**Table 1:** Basic patient characteristics.

	N = 1183
Age	
≤50	102 (8.6%)
51–65	379 (32.0%)
66–80	522 (44.1%)
>80	180 (15.2%)
Performance status	
ECOG	n = 276
0	119 (43.1%)
1	102 (37.0%)
2	28 (10.1%)
3	20 (7.3%)
4	7 (2.5%)
Unknown	n = 907
Histology	N = 1183
HGSOC	781 (66.0%)
Adenocarcinoma	162 (13.7%)
Other	105 (8.9%)
Unspecified	135 (11.4%)
Metastasis localization	
Known	n = 954
Unknown	n = 229
Pleura	309 (32.4%)
Lymph nodes	225 (26.7%)
Liver	278 (29.1%)
Lung	104 (10.9%)
Other	231 (24.21%)
Brain	10 (1.1%)
Generalized	6 (0.6%)
Peritoneum	95 (10.0%)
Skin	16 (1.7%)
Adrenal gland	10 (1.1%)
Bone	42 (4.4%)
Bone marrow	1 (0.1%)

**Table 2:** Basic treatment data.

	N = 1183
Treatment received	
None	221 (18.7%)
Surgery only	145 (12.3%)
Systemic only	279 (23.6%)
Radiation only	5 (0.4%)
Surgery + systemic	473 (40.0%)
Surgery + radiation	1 (0.1%)
Surgery + radiation + systemic	50 (4.23%)
Systemic + radiation	9 (0.8%)
Received surgery	669 (56.6%)
Received systemic	811 (68.6%)
Received radiation	65 (5.5%)
Intention of first surgery	(n = 669)
Diagnostic	48 (12.0%)
Curative	220 (55.0%)
Palliative	113 (28.3%)
Revision	3 (0.8%)
Other	16 (4.0%)
Unknown	269
Residual status after first surgery	(n = 669)
R0	157 (37.1%)
R1	118 (27.9%)
R2	148 (35.0%)
RX/Unknown	246
Sequence of surgery and systemic therapy, disregarding radiation therapy	n = 1183
No surgery, no systemic	226 (19.1%)
Surgery only	146 (12.3%)
Surgery followed by systemic	452 (38.2%)
Systemic only	288 (24.3%)
Systemic followed by surgery	71 (6.0%)
Primary treatment within 2 months post-diagnosis	n = 1183
No surgery, no systemic	226 (19.1%)
Surgery	577 (48.8%)
Systemic	305 (25.8%)
Surgery/systemic later	75 (6.3%)
Treatment scheme *	n = 1183
Surgery only	119 (10.0%)
Surgery followed by systemic	376 (31.8%)
Systemic	267 (22.6%)
Systemic only	201
Systemic followed by surgery	66
other	421 (35.6%)

* Restrictions on minimum follow-up time after primary treatment were set to avoid any time-dependent bias. Surgery only: surgery was performed within 2 months post-diagnosis, and no further systemic therapy was performed within 3 months thereafter. Surgery followed by systemic: surgery was performed within 2 months post-diagnosis and systemic therapy was started within 3 months thereafter. Systemic: systemic therapy was started within 2 months post-diagnosis, and follow-up was available at least 3 months thereafter.

**Table 3:** Treatment of patients by age, performance status, histology and metastasis localization: sequence of surgery and systemic treatment.

	No Surgery/No Systemic Treatment	Surgery Only	Surgery Followed by Systemic	Systemic Treatment Only	Systemic Followed by Surgery
	N = 226	N = 146	N = 452	N = 288	N = 71
Age (N = 1183)					
≤50 (N = 102)	15 (14.7%)	8 (7.8%)	48 (47.1%)	23 (22.6%)	8 (11.3%)
51–65 (N = 379)	48 (12.7%)	37 (9.8%)	183 (48.3%)	86 (22.7%)	25 (6.6%)
66–80 (N = 522)	94 (18.0)	65 (12.5%)	192 (36.8%)	135 (25.9%)	36 (6.9%)
>80 (N = 180)	69 (38.3%)	36 (20.0%)	29 (16.1%)	44 (24.4%)	2 (1.1%)
ECOG (N = 276)					
0 (N = 119)	11 (9.2%)	19 (16.0%)	67 (56.3%)	18 (15.1%)	4 (3.4%)
1 (N = 102)	11 (10.8%)	17 (16.7%)	42 (41.2%)	26 (25.5%)	6 (5.9%)
2 (N = 28)	9 (32.1%)	4 (14.3%)	8 (28.6%)	7 (25.0%)	0
3 (N = 20)	6 (30.0%)	6 (30.0%)	5 (25.0%)	3 (15.0%)	0
4 (N = 7)	5 (71.4%)	1 (14.3%)	0	1 (14.3%)	0
Unknown (N = 907)	184	99	330	233	61
Histology (N = 1183)					
HGSOC (N = 781)	111 (14.2%)	108 (13.8%)	340 (42.5%)	164 (21.0%)	58 (7.4%)
Adenocarcinoma (N = 162)	44 (27.2%)	13 (8.0%)	54 (33.3%)	44 (27.2%)	7 (4.3%)
Other (N = 105)	18 (17.1%)	16 (15.2%)	39 (37.1%)	29 (27.6%)	3 (2.9%)
Unspecified (N = 135)	53 (39.3%)	9 (6.7%)	19 (14.1%)	51 (37.8%)	3 (2.2%)
Metastasis localization (N = 954)					
Pleura (N = 309)	52 (16.8%)	39 (12.6%)	118 (38.2%)	75 (24.3%)	25 (8.1%)
Lymph nodes (N = 255)	43 (16.9%)	20 (7.8%)	106 (41.6%)	72 (28.2%)	14 (5.5%)
Hepatic (N = 278)	58 (20.9%)	23 (8.3%)	97 (34.9%)	89 (32.0%)	11 (4.0%)
Lung (N = 104)	29 (27.9%)	14 (13.5%)	23 (22.1%)	33 (31.7%)	5 (4.8%)
Others (N = 369)	72 (19.5%)	57 (15.5%)	137(37.1%)	87 (23.66%)	16 (4.3%)
Unknown (N = 229)	42	33	88	50	16

**Table 4:** Analysis of overall survival.

(A) Age and histology.						
	Hazard Ratio		95%-Confidence Interval		*p*-Value	
Age					<0.0001	
≤50	1.00		-			
51–65	1.14		[0.84, 1.55]			
66–80	1.92		[1.41, 2.56]			
>80	3.81		[2.76, 5.27]			
Histology					<0.0001	
HGSOC	1.00		--			
Adenocarcinoma	1.49		[1.21, 1.82]			
Other	1.52		[1.20, 1.93]			
Unspecified	1.47		[1.18, 1.82]			
(B) Comparison of treatment groups.						
	Unadjusted Analysis		Adjusted for Age and Histology			
	Hazard Ratio	95%-Confidence Interval	*p*-Value	Hazard Ratio	95%-Confidence Interval	*p*-Value
Primary treatment within 2 months post-diagnosis (N = 882) Surgery vs. Systemic	0.82	[0.68, 0.97]	0.025	0.90	[0.75, 1.08]	0.27
Treatment scheme * (N = 762) Surgery only vs. Systemic Surgery followed by systemic vs. Systemic Surgery followed by systemic vs. Surgery only	0.79 0.72 0.92	[0.59, 1.05] [0.59, 0.86] [0.70, 1.21]	0.007	0.81 0.81 1.01	[0.60, 1.08] [0.66, 1.00] [0.76, 1.33]	0.12

* This analysis only included patients who started treatment within 2 months after initial diagnosis, and for whom the minimum follow-up was at least 3 months after the primary treatment, to avoid any time-dependent bias.

## Data Availability

The data presented in this study are available on request from the corresponding author.
